# Theory-Based Interventions in Physical Activity: A Systematic Review of Literature in Iran

**DOI:** 10.5539/gjhs.v7n3p215

**Published:** 2014-11-30

**Authors:** Jalal Abdi, Hassan Eftekhar, Fatemeh Estebsari, Roya Sadeghi

**Affiliations:** 1 Department of Health Education and Promotion, School of public Health, Tehran University of Medical Sciences, Tehran, Iran; 2 School of Public Health, IRAN University of Medical Sciences, Tehran, Iran

**Keywords:** health education interventions, Iran, model, physical activity, theory

## Abstract

Lack of physical activity is ranked fourth among the causes of human death and chronic diseases. Using models and theories to design, implement, and evaluate the health education and health promotion interventions has many advantages. Using models and theories of physical activity, we decided to systematically study the educational and promotional interventions carried out in Iran from 2003 to 2013.Three information databases were used to systematically select papers using key words including Iranian Magazine Database (MAGIRAN), Iran Medical Library (MEDLIB), and Scientific Information Database (SID). Twenty papers were selected and studied. Having been applied in 9 studies, The Trans Theoretical Model (TTM) was the most widespread model in Iran (PENDER in 3 studies, BASNEF in 2, and the Theory of Planned Behavior in 2 studies). With regards to the educational methods, almost all studies used a combination of methods. The most widely used Integrative educational method was group discussion. Only one integrated study was done. Behavior maintenance was not addressed in 75% of the studies. Almost all studies used self-reporting instruments. The effectiveness of educational methods was assessed in none of the studies. Most of the included studies had several methodological weaknesses, which hinder the validity and applicability of their results. According to the findings, the necessity of need assessment in using models, epidemiology and methodology consultation, addressing maintenance of physical activity, using other theories and models such as social marketing and social-cognitive theory, and other educational methods like empirical and complementary are suggested.

## 1. Introduction

Physical inactivity is now identified as the fourth leading risk factor for global mortality (World Health Organization [WHO], 2010). Physical inactivity levels are rising in many countries with major implications for the prevalence of non communicable diseases (NCDs) and the general health of the population worldwide, about 3.2 million people die due to lack of physical activity Every year (WHO, 2013, 2010).

With changing social and economic patterns all over the world, sedentary lifestyles have become a worldwide phenomenon (Lee, Macfarlane, Lam, & Stewart, 2011). This phenomenon is common in teenagers, adults, and elderly people worldwide (Timori & Esmailnasab, 2011).

Despite all significant effects on human life, technology has increased the tendency to inactive lifestyles (karimi & Eshrati, 2012). Today, lack of physical activity is considered as one of the most important problems in the field of public health (Solhi, Motlagh, Shirazi, Taghdisi, & Jalilian, 2012; Tabatabaei, Taghdisi, Sadeghi, & Nakhaei, 2010).

There is a wide consensus on the benefits of physical activity in health and in disease (Oyeyemi et al., 2011). Regular physical activity has an important role in reducing the risk of diseases such as cardiovascular disease, diabetes, cancer, and in weight management to prevent obesity (Van Wier et al., 2006). Physical activity (PA) reduces the rate of hospitalization, visiting doctors, and the need to take medicine. It can also reduce the rate of absence from work and accordingly increases the productivity and engaging in job. Promoting mental health, self-esteem, mood, and reducing the risk of stress and depression are among the benefits of physical activity (WHO, 2013; Pirasteh et al., 2012; Strijk, Proper, van Mechelen, & van der Beek, 2013).

Physical inactivity is common in Iran, particularly in females and in the older age groups (Esteghamati et al., 2011). Koohpayehzadeh et al. (2014) showed that the overall prevalence of physical inactivity in Iran was increased from 15% (2007) to 21.5% (2011) Over the 4 years, 56.4%, 39.2%, and 74.4% of participants were physically inactive at work, commuting and recreation, respectively.

It has been evidently shown that 70-80% of people in Iran lack enough physical activity (Solhi, Ahmadi, Taghdisi, & Haghani, 2011; Hashemi, Rakhshani, Navidian, & Mosavi, 2013) and 65% of youth are far from reaching the recommended levels of physical activity [i.e., 30 min of moderate PA per day, five or more days of the week or vigorous PA at least 20 min per day, three or more days a week] (Saffari, Amini, Ardebili, Mahmoudi, & Sanaeinasab, 2012b).

The world is shifting towards the use of theory- based interventions to increase the level of PA. Glanz and Bishop (2010) stated that, “Increasing evidence suggests that public health and health-promotion interventions that are based on social and behavioral science theories are more effective than those lacking a theoretical base.” While little success in changing behavior is documented where no theory was used, theory-based interventions have had significant success in designing effective interventions that are guided by constructs of each theory to change people’s behaviors. Theory-based interventions have been associated with larger and longer-term effects than those without an explicit basis in theory (Skaal & Pengpid, 2012). The most common Theories/models in health education/promotion programs are presented in Appendix A.

Several studies have confirmed the effect of education on changing physical behavior (WHO, 2012; Moeini et al., 2010; Saffari, Shojaeezade, Ghofrani Pour, Heydarnia, & Pakporhajiagha, 2012a). To improve the effectiveness of health education programs, it is proposed to use patterns and theories. Each model or theory follows a structural sequence of planning, implementation, and evaluation. Applying these models and theories 1: helps to identify the measurable sequences of the program, 2: reveals how behaviors go through changes, 3: determines the timing of interventions, 4: helps to choose an appropriate combination of strategies, 5: improves the relationship between experts, and 6: improves the programs repetitions and boosts the effectiveness of the program (Saffari et al., 2012a).

In recent years, health education models and theories have been increasingly used in Iran as a framework for designing and implementing interventions. The aim of this review was to critical appraisal of the existing literature reported on the effectiveness of theory-based interventions in physical activity in Iran.

## 2. Methods

### 2.1 Search Strategy

In order to identify studies in which health education and promotion models and theories were applied, three databases including, MAGIRAN, MEDLIB, and SID were searched. These database publish scientific and peer review studies.

Studies available on these websites were selected according to (1) physical activity (2) health behavior change model (3) health education and promotion models (e.g: BASNEF), (4) the aim of the intervention (e.g: identifying the effectiveness of education), and (5) the type of the study (e.g:clinical trial and PRECEDE).

All studies were performed from 2003 to 2013. Before 2003, there were no articles that used health education and promotion models/theories in physical activity.

### 2.2 Selecting the Studies

The title and the abstract of the studies were saved on an electronic database for future references and reference management all articles were selected by two authors, independent of each other possible disagreement were resolved by discussion and consensus. After an initial list of included studies was developed, the third author, an expert in health education and health promotion reviewed the list for completeness.

### 2.3 Inclusion Criteria

In order for a study to be selected, 5 criteria were defined:

Studies that used health education and promotion models and theories.

Studies that aimed to investigate the effect of education using theories and models.

Studies that were experimental or a quasi- experimental.

Studies that yielded physical activity as one of its main outcomes.

Studies that were published in Persian.

No limits were considered for the type of intervention, the duration of intervention, and follow-up activities. (Flow chart 1)

**Flowchart 1:**
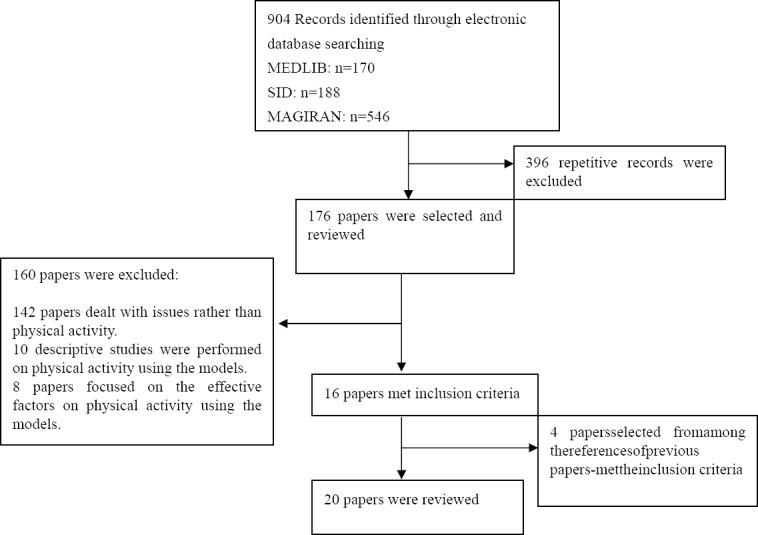
Stages of selecting and reviewing papers

### 2.4 Exclusion Criteria

Studies that were descriptive and cross-sectional.

Studies that used health education and promotion models/theories to simply investigate effective factors and determinants in physical activity.

### 2.5 Data Collection, Data Analysis, and Classification

To collect required data, 1) selected studies were scrutinized(rough plan, the duration of the study, etc.), 2) the features of the population were evaluated(job characteristics, etc.) and 3) the focus of the study (physical activity promotion), and 4) the way of measuring outcomes were studied (self reporting, using the instruments, etc.).

### 2.6 Quality Assessment Tool

We adapted methodological quality assessment for the included studies from CONSORT (Brooks, Higgins & Webster, 2010) and Chen et al, s check list (2014). A total Methodological quality score was calculated by summing up all “yes” studies that met 70% of the criteria, were rated as having high methodological quality (Table 3).

**Table 1:** The results of the systematic review

	Maintenance of behavior	Results	The method of education	Duration of intervention	The typeof the study^*^	Target group	Model/theory	The aim of the study	Author(s)	
1	Jalilian et al. (2013)	the effectiveness of TTM-based educational intervention in promotion of regular physical activity among the staff	TTM	staff	Quasi-Experimental	3 months	Pamphlets, booklets, CDs	significant progress during the stages of change; significant increase in TTM constructs, and in the level of awareness; counter conditioning; motivation control; self-libration and helping relationships	-
2	Farmanbar et al. (2011)	the impact of intervention on promotion and maintenance of athletic behavior based on an integration of TTM and self-determination theory	integration of TTM and self-determination theory	university students	RCT	before the study, during the study, and 8 months afterthe study	group discussion, public meetings, 4 educational sessions (45-60minuts), CDs, booklets, brochures, SMS, reminder cards	changes in the level of athletic behavior and post-intervention stages of change	significant increase in the average athletic behavior in the interventiongroup 8 months after the intervention versus during the study; increase in the number of people inthe preparation stage, decrease in the number of people in the stage of action, increase in the number of people in the stage of maintenance; decrease in the index self-determination, understanding of autonomy and belonging
3	Moeini et al. (2012)	the impact of 8 weeks of educational intervention on promotion of physical activity in diabetic patients	TTM	diabetic patients	Quasi-Experimental	before the intervention and one month after (8 weeks of intervention)	lecture, discussion, pamphlet, booklet, CDs, work out	increase in the stages of change in exercising; increase in the average ofthe constructs of cognitive and behavioral processes as well as in self-efficacy; significant difference in applying the levels of processes of cognitive and behavioral change	
4	Solhi et al. (2011)	determination of the impact of applying TTM on physical activity	TTM	pregnant women	before and after the study	lectures (5 one-hour sessions); group discussion; showing educational films	promotion in the stages of change (from pre-contemplation to contemplation and to the preparation stage); increase in the average of perceived benefits, perceived barriers, perceived related pleasure, and perceived social support; increase in physical activity	
5	Hashemi et al. (2013)	determination of the impact of education on the level of physical activity in pregnant women	TTM	pregnant women	controlled interventional	2 months	discussing the goals, booklet, educational cards, educational strategies to draw attention, motivational interviews, self-efficacy promotion, motivating strategies, consultation to overcome temptation situations and anxiety, consultation on the risk of attention deficit, self-rewarding	significant increase in the average of awareness score, attitude, and model constructs in the intervention group (perceived benefits, perceived barriers, and -self-efficacy)	
6	Moeini et al. (2010)	the impact of physical activity educational programs on the promotion of physical activity and increasing physical strength	TTM	staff	Quasi-Experimental	12 weeks	lecture, group discussion, workshop, pamphlet, booklet	Significant progress in the stages of change; increase in the level of physical strength, self-efficacy, balance, and sport activities	-
7	Ghahremani et al. (2008)	application of TTM structures to promote physical activity in the elderly	TTM	the elderly	experimental	2 months	individual consulting sessions; group discussion; 8 educational sessions (three 10-15 minutes private sessions for 3 weeks and three 45-60 minutes sessions of group discussion; 2 sessions of workout; sport coach and physiotherapists	Significant difference in the stages of preparation; creating balance in decision making and physical activity behavior in the elderly	-
8	Karimzade et al. (2007)	determination of the impact of education on the promotion of physical exercise level, muscular strength, and balance	TTM	women between 40-65	experimental	12 weeks	implementing educational program in two parts (public and private) using lectures, films, slideshows, group discussions, self-assessment, pamphlets, practical education, drawing attention strategies	Significant progress in the stages of change; increase in the duration of physical activity, muscular strength, dynamic and static balance	-
9	Mardanihamole et al (2010)	Impact of educational program based on TTM on PA	TTM	Patient with IBD	Quasi-experimental		Individual consulting(3 session), GD (3 session)	Significant increase in PA, self efficacy and decisional balance	----
10	Sollhi et al. (2012)	designing and implementingan educational program to promote physical activity	TPB	school students	experimental	before and 2 months after the intervention	emphasis on dynamic learning, lectures, group discussion, peer education method, pamphlets, booklets, and CDs	Significant difference in the average score of attitude, behavioral intention, subjective norms and perceived behavioral control, increase in physical activity	-
11	Tabatabaee et al. (2010)	determination of the impact of educational intervention on the level of physical activity inthe staff	TBP	staff	Quasi-experimental	6 weeks	placing motivating manuscripts on the boards inthe workplace; lectures and PowerPoint presentations; pamphlets; issuing certificates after passing the refreshment course; disseminating photos and messages through Bulk Messaging systems every 5 days; disseminating motivating messages weekly	Significant increase in the average scores of awareness and behavioral intention in the intervention group; decrease in the score of perceived control; lack of Significant change in the average physical activity and subjective norm construct	
12	Estebsari et al. (2010)	determination of the impact of education on physical activity promotion on the basis of PRECEDE Method	PRECEDE	junior high school students	Quasi-experimental	before and 2 months after the intervention	lectures, free discussion, pamphlets, films, essay writing and newspaper writing contests	increase in the average score of predisposing, enabling, and reinforcing factors; improving awareness and attitude; promotion in physical activity	-
13	Saffari et al. (2012)	evaluation ofthe impact of educational intervention onthe life style based on the PRECEDE model	PRECEDE	youth	randomized controlled trial	before and 2 months after the intervention	educational package, 5 one-hour sessions	increase in the average score of standardized physical activity in boys rather than girls	
14	Teimori et al. (2007)	determination of the impact of school interventions on physical activity promotion	PENDER	students	trial	24 weeks	education in groups of 5/7/12 students for 30-45 minutes; short lectures; slideshows; films; group discussion; question and answer meetings; role-play; individual consultation; reminder cards; pamphlets	increase in the amount of time devoted to daily physical activity; progress in the stage of preparation caused by intervention; development in perceived self-efficacy and perceived behavior-related pleasure; inter-personal effects; having plans for actions; decrease in the perceived barriers and competitive preferences	-
15	Karimi et al. (2011)	determination of the impact of education on the promotion of physical activity	PRNDER	university students	before and after random controlled	two months	six sessions (lecture and group discussion); targeting and planning strategies to create motivation	Significant difference in the average scores of variables inthe Health Promotion Model (perceived benefits and barriers, self-efficacy, inter-personal effective factors); better performance at the end of the program	-
16	Noroozi et al. (2011)	studying the impact of education on physical activity	PENDER	diabetic women	Quasi-experimental	at the beginning of the study, 3 months and 6 months after the study	group education (one session); private consultation usingthe 5A method (3 sessions)	change in the level of physical activity; difference in the levels of activity betweencases	increase in the number of individuals in the action and maintenance stages as well as decrease in the level of physical activity in the 6^th^ month (in comparisonwith the 3^rd^ month)
17	Hazave’ei et al (2009)	the impact of PE(2) on regular physical activity based on the BASNEFmodel	BASNEF	female university students	experimental	before, and 2 and 4 months after	8 educational sessions (30 minutes) lecture, film, question and answer, discussion, pamphlets, CDs, answering the students’ questions via telephone	better performance ofthe intervention group in physical activity 2 and 4 months after the interventions; significant change inthe average scores of BASNEF constructs (enabling factor, subjective norms, attitude toward regular physical activity, attitude toward the outcomes of regular physical activity)	decrease in the score of awareness, attitude toward outcomes, enabling factors, and intention in first and second follow-up than the immediate stage after intervention; decrease in the score of attitude in the first follow-up and increase in the second follow-up; increase in determined decision to do physical exercise in the first and second follow-up than the immediate stage after the intervention
18	Shakeri et al. (2012)	determination of the impact of educational intervention on the level of physical activity in pregnant women based on the BASNEF Model	BASNEF	pregnant women	quasi-empirical	before, and immediately, and 6 weeks after the intervention	CDs, pamphlets, booklets, lectures, group discussion. films, question and answer sessions, work out in groups in the presence of an expert	difference in the scores of attitude, awareness, enabling factors, and subjective norms; promotion of physical activity in the group	increase in the score of knowledge, attitude, subjective norms, and enabling factors in the intervention group
19	Peyman et al. (2012)	determination of the impact of education on self-regulation strategies	self-regulation	diabetic women	randomized controlled clinical trial	before, after and 10 weeks after the intervention	7 one-hour sessions (4 theoretical sessions and 3 sessions of workout), group discussion, brain storming, lectures, posters, pamphlets, fact sheets, DVD	Significant difference in targeting, planning, awareness and the level of physical activity; significant decrease in the level of blood sugar and body mass index	decrease in the scores of targeting, planning, and awareness in follow-up stages than the stage after intervention; decrease in the level of physical activity in the follow-up stage
20	Abedi et al. (2011)	determination of the impact of Health Belief Model on the cardiovascular risk factors	Health Belief Model	menopausal women	clinical trial	before and 6 months after the intervention	One face-to-face educational session; one session at the end of the third month; three one-hour sessions using aid kits in the first month and at the end of every week, pamphlet,	increase in physical activity (272 minutes a week); increase in knowledge, perceived severity, and perceived susceptibility	-

**Table 2:** Dirubtion of Educational Methods in Physical activity Theory Based interventions in Iran

Reinforcement Methods																													

SMS		Pamphlet		poster		DVD&CD		brochure		booklet		reminder cards		Lecture		Answering &questioning		placingmotivating mottos on the boards&Bilbord		Fact Sheets &Tract		film		Slide		Motivational Interview		consulting	

%	N	%	N	%	N	%	N	%	N	%	N	%	N	%	N	%	N	%	N	%	N	%	N	%	N	%	N	%	N
**10**	**2**	**35**	**7**	**5**	**1**	**30**	**6**	**35**	**7**	**30**	**6**	**15**	**3**	**65**	**13**	**35**	**7**	**5**	**1**	**5**	**1**	**30**	**6**	**10**	**2**	**5**	**1**	**20**	**4**
**experiential methods**															**Integrative Methods**														
Role Playing								Workshop							Brain Storming								Group Discussion						
%				N				%				N			%				N				%				N		
**25**				5				5				1			5				1				70				14		

**Table 3:** Methodological quality of the included studies

Reference	Randomization	Blinding	Inclusion/Exclusion criteria clearly described	Adequate sample size calculation shown	Standard measures described	Maintenance	Situation analysis and reasons for selecting Theory/model explained	Rational for Duration/Dose of intervention	score
Karimi et al. (2012)	1	0	1	0	1	0	1	0	4/8
Tabatabaei et al. (2010)	1	1	0	0	1	0	1	0	4/8
Abedi et al. (2011)	1	0	1	0	1	0	NA	0	3/8
Hazaveei et al. (2009)	1	0	1	0	1	1	0	0	4/8
Jalilian et al. (2012)	1	0	0	0	1	0	1	0	3/8
Moeini et al. (2010)	1	0	1	0	1	0	1	0	4/8
Hashemi et al. (2013)	1	0	1	1	NA^2^	0	1	0	4/8
Solhi et al. (2011)	1	0	1	0	NA	0	1	0	3/8
Shakeri et al. (2012)	1	0	1	1	1	1	0	0	5/8
Timori et al. (2007)	1	0	1	0	1	0	1	1	5/8
Peyman et al. (2012)	1	0	1	0	1	1	1	0	5/8
Saffari et al. (2012b)	1	0	1	1	1	0	0	1	5/8
Solhi et al. (2012)	1	0	1	1	1	0	1	1	6/8
Estebsari et al. (2010)	1	0	0	1	1	0	0	0	3/8
Noroozi et al. (2011)	1	0	1	0	1	1	1	1	6/8
Farmanbar et al. (2001)	1	0	1	0	1	1	1	1	6/8
Moeini et al. (2011)	1	0	1	0	1	0	1	1	5/8
Mardanihamole et al. (2010)	1	0	1	0	1	0	1	1	5/8
Karimzadehshirazi et al. (2007)	1	0	1	0	1	0	1	1	5/8
Ghahremani et al. (2008)	1	0	1	0	1	0	0	1	4/8

## 3. Results

All studies except for one case(Tabatabaei et al., 2010) showed that target intervention was effective in the promotion of physical activity. The most commonly used model in physical activity was the trans theoretical model (TTM) which was used in 9 studies. Other models and theories which were applied in the studies were the PENDER model (3 studies), the PRECEDE model (2 studies), the theory of planned behavior (2 studies), the BANSEF model (2 studies), the self regulation theory (1 study), and the health belief model [HBM] (1 study). Regarding the type of the study, all studies were experimental, except for 8 quasi- experimental studies (Table 1). Of 20 trials, only 3 trials provided evidence of high quality. The sample size varied in different studies, ranging from 25 to 300 people. Five studies addressed maintenance of physical activity (performed in different periods of time) [Hazavehei et al, 2009; Shakeri et al., 2012; Farmanbar et al, 2011; Peyman et al, 2012 & Noroozi et al, 2011]. Approximately 65% of the studies explained the background and their reasons for selecting the model (Table 3). With regards to the educational methods, almost all studies used a combination of methods; 65%used lectures and 35% used pamphlets. The peer education method and motivational interviewing were used in only one study (Solhi et al., 2012). As for the experiential methods, role playing was used in five and workshop in one study. The most important integrative method used in the studies was group discussion (14 studies). None of the studies measured the effectiveness of the educational method (Tables 1 and 2).

## 4. Discussion

The present review investigated the theory based intervention in PA in Iran and the effect of these interventions.

### 4.1 Main Results

Approximately all the studies conducted in Iran focused on the individual or intrapersonal level, and individuals were the primary target audience of the health education materials.

All studies except for one case stated that target intervention was effective in the promotion of physical activity.

The studies were heterogeneous and had different sample sizes (ranging from 25-300). All the studies relied on self-reporting. The interventions differed in target populations, duration of intervention, and settings. Of 20 trials, only 3 provided evidence of high quality.

Almost all studies used a combination of methods. PA maintenance was not addressed in 75% of the studies.

### 4.2 Summary of the Lessons Learned From This Review

Most of the studies that conducted in Iran had methodological weaknesses.

In30-40% of the studies, the reasons for choosing the theories/models were not specified.

The social and ecological models were not addressed, Although social marketing and social-cognitive theory are applicable models/theories in physical activity, we could not find any records that were designed and implemented based on these models/theories in Iran.

### 4.3 Theoretical Basis for Intervention

Of nine studies based on TTM, all reported positive results in the treatment group. When compared to the control group, the treatment group had a significant progress during the stages of change and TTM constructs (Table 1).

Of two studies based on TPB, Tabatabaee et al. (2010) reported that TPB did not lead to an increase in physical activity. Azjen and Fishbein (2004) showed that the predictability of TPB differed from behavior to behavior and from population to population.

In Iran, only three studies had the inclusion criteria of PENDER.all of these studies had some positive results.

In studies performed by Saffari et al. (2012b); Estebsari et al. (2010); Pyman et al. (2012) and Abedi et al. (2011), the theory/model was partially implemented.

Only two studies contained BASNEF inclusion criteria. They did not deal with the application of BASNEF model in physical activity studies, but the maintenance of physical activity was addressed in studies by Hazavehei et al. (2009), 4 months after the interventions and Shakeri et al (2012), 6 weeks after the intervention.

### 4.4 Overall Completeness and Applicability of Evidence

Of twenty trials, 7 were conducted in the school/university and 3 in the workplace. the others were conducted in clinical settings. Most studies included promotion of PA as a main objective. All of them provided multiple sessions. One trial focused on elderly people. Almost all studies used a combination of methods.

Methods are the means or ways that we use to deliver the material to our clients. In a classification, educational methods are divided into three categories: 1) reinforcement methods 2) integrative methods and 3) experiential methods (Saffari et al, 2012a). Most educators agree that reinforcement methods such as lectures are necessary (Saffari et al., 2012a; Tyler et al., 2009), but they believe that they should be limited in number and well delivered. It is important to give the learners the opportunities to apply and reflect on lecture material during course time.Our findings showed that the most important educational methods that were used in the studies were the reinforcement method (65% used lectures). Lectures are efficient ways of delivering information. but the weaknesses of lecture should be considered.

Traditionally, small groups consist of 8-12 participants. Small groups can take on a variety of different tasks, including problem solving, role play, discussion, brainstorming, debate, workshops, and presentations. In our study, the most widely used integrative educational method was group discussion.

The main advantages of small group learning are that it encourages active learning and develops communication and teamwork skills.

The peer education method and motivational interviewing were used in only one study.

### 4.5 Limitations and Quality of the Evidence

Of 20 trials, only 3 provided evidence of high quality (Table 3).

All the studies reviewed here relied on self-reporting (except for Moeini et al, 2010). The reliability of self-reporting is questionable. Because of social desirability and other types of information bias, self report is not the most reliable indicator of behavior (Lopez, Tolley, Grimes, Chen, & Stockton, 2013).

Choosing a theory should start with a “thorough assessment of the situation: the units of analysis or change, the topic, and the type of behavior to be addressed” (WHO, 2012). We found that, some studies specifically stated their reasons for selecting the theory/model, while, many studies did not provide sufficient information about the assessment of the situation.

Sample size calculation was poorly reported. Only one trial provided blinding of participants (Tabatabaei et al., 2010). The flow of participants was reported in none of the studies. Randomization details were frequently unclear.

Because of the variation in the duration of intervention(ranging from immediately to 24 week) the studies that conducted in Iran does not provide enough evidence on the optimal duration of most effective intervention for promoting physical activity. This is consistent with a systematic review performed by Chen and Wilkosz (2014).

Effectiveness may be limited when the theory/model is partially implemented (Lopez et al., 2013). Peyman et al. (2012), used only two components of the self-regulation theory (SRT), e.g. setting goals and perusing goals. Other components of SRT were not addressed in their study. As Saffari et al. (2012b) mentioned, some stages of PRECEDE (such as genetic diagnosis) could be ignored. Estebsari et al. (2010), evaluated the stages of educational diagnosis (predisposing, enabling, reinforcing).

Constructs such as awareness, perceived susceptibility, perceived severity, perceive benefits, and perceived barriers were evaluated in a study by Abedi et al. (2011). A recent addition to the HBM is the concept of self-efficacy, which was added to the model in 1980. Bendura reported that self-efficacy was one of the most important structures in such behaviors as physical activity (Moeini et al., 2010; Jallilian et al., 2013). In the study by Abedi et al. (2011), self-efficacy was not evaluated.

#### 4.5.1 TTM

Our Findings show that TTM is frequently used in physical activity studies in Iran. In spite of its popularity, TTM has its own limitations. First, the reliability of self-reporting is questionable. All the studies reviewed here relied on self-reporting. Only Moeini et al. (2010) used Ergo line bicycle to evaluate the physical strength in the sample population. Second, the TTM lacks predictability. It has not been addressed in the studies published in Iran. Some scholars, however, consider this model as a descriptive, not a predictive one (Sharma & Romas, 2008).

Our systematic review revealed that several questionnaires were used to measure the constructs of the TTM.For example, Jalilian et al (2013) used 5 questionnaires. Being economical is considered as one of the characteristics of a good theory or model and the TTM is not an economical model (Saffari et al., 2012a). Another limitation of the TTM is that people can easily pass stages or return to the previous stages. No study in Iran ever dealt with the issue of returning to previous stages.

#### 4.5.2 TPB

One of the limitations of TPB is that it assumes that perceived behavioral control can predict actual behavior control. It is confirmed by the decrease in perceived behavior control observed in the study performed by Tabatabaei et al (2010). Measurement of intention requires measurement of its predictors which in the context of TPB is most commonly inferred from questionnaire responses and measuring behavior using self-reporting is another limitation of TPB. Self-reporting has been used as a measure in TPB-based studies in Iran. Ajzen and Fishbein (2004) reported that “such behaviors as physical activity were time-consuming and expensive to study” (Saffari et al., 2012a).

#### 4.5.3 PENDER

One of the main disadvantages of this model is that it contains too many constructs, and is not economical. For example, Teimori et al (2007) used 7 questionnaires. It does not seem economical or easy to control. It is also quite time-consuming (Saffari et al, 2012a).

#### 4.5.4 Other Models

Implementing PRECEDE needs significant financial sources. According to Saffari et al (2012b), it is usually impossible to evaluate outcomes in this model.

Since HBM focuses on a limited number of factors and ignores cultural, social, and economical factors and the previous experiences of people, it essentially lacks predictability (Saffari et al., 2012a).

One of the main challenges in health education and promotion is the maintenance of the behavior which was considered in only 5 studies in different periods of time without any explanation on the reason.

## 5. Conclusion

This review can be used to design and implement theory/model based interventions in physical activity, but the methodological weaknesses among the studies (e.g lack of sample size adequacy, variation in the duration of intervention, lack of rationale for selecting models/theories, etc …) should be considered. Limitations and weaknesses listed above could affect the validity and applicability of the results of these studies.

Considering the findings of the study, the authors suggest that:

Maintenance in PA and integrating models in the field of physical activity should be addressed;

Future researches should include long term follow ups, longer intervention periods, and larger sample sizes to evaluate the effectiveness of theory -based interventions in PA.

Health promotion programs are more effective when planners consider multiple levels of influence on health problem. Lack of addressing interpersonal and community levels in Iranian trials is important and should be included in future research. Moreover, epidemiology methodology consultation is necessary.

The reliability and validity of assessment tools in Iranian studies must be one of the important priorities. Some other effective social and ecological models in the field of physical activity are social marketing and social-cognitive theory which were not performed in Iran. It is suggested that future studies include these models.
